# Examining the Phenomenon of Quarter-Life Crisis Through Artificial Intelligence and the Language of Twitter

**DOI:** 10.3389/fpsyg.2020.00341

**Published:** 2020-03-06

**Authors:** Shantenu Agarwal, Sharath Chandra Guntuku, Oliver C. Robinson, Abigail Dunn, Lyle H. Ungar

**Affiliations:** ^1^Department of Computer and Information Science, University of Pennsylvania, Philadelphia, PA, United States; ^2^Department of Psychology, Social Work and Counselling, University of Greenwich, London, United Kingdom; ^3^Department of Psychology, University of Sussex, Brighton, United Kingdom

**Keywords:** quarter-life crisis, machine learning, natural language processing, social media, emerging adulthood

## Abstract

Quarter-life crisis (QLC) is a popular term for developmental crisis episodes that occur during early adulthood (18–30). Our aim was to explore what linguistic themes are associated with this phenomenon as discussed on social media. We analyzed 1.5 million tweets written by over 1,400 users from the United Kingdom and United States that referred to QLC, comparing their posts to those used by a control set of users who were matched by age, gender and period of activity. Logistic regression was used to uncover significant associations between words, topics, and sentiments of users and QLC, controlling for demographics. Users who refer to a QLC were found to post more about feeling mixed emotions, feeling stuck, wanting change, career, illness, school, and family. Their language tended to be focused on the future. Of 20 terms selected according to early adult crisis theory, 16 were mentioned by the QLC group more than the control group. The insights from this study could be used by clinicians and coaches to better understand the developmental challenges faced by young adults and how these are portrayed naturalistically in the language of social media.

## Introduction

Quarter-life crisis (QLC) is a phenomenon that has become widely discussed in the media and in popular writings about the challenges of early adulthood (Robbins and Wilner, 2001; Rosen, 2019). Academic discourse on the phenomenon has also developed in the form of empirical and theoretical work on developmental crisis episodes during the first decade of adult life (Robinson, 2016) and also in applied disciplines such as coaching (Stapleton, 2012). The present study explores how the phenomenon is linguistically rendered in the social media space of Twitter, with the aim of better understanding the popular conception of the phenomenon and how that may help to convey the nature of key developmental challenges pertaining to being a young adult.

The theoretical frameworks used to analyze, explore, and interpret QLC are the theory of emerging adulthood (Arnett, 2000, 2007) and the model of early adult crisis by Robinson and Smith (2010), Robinson et al. (2013), and Robinson (2019). The theory of emerging adulthood proposes five defining developmental features of the age range of 18–28. These are: (1) feeling ambiguous in terms of adult status – young people in this age range typically describe themselves as in some ways an adult, yet in some ways not, and as being caught in between; (2) a period of active exploration of self and world; (3) a time of instability in roles and relationships, stemming from a continued lack of long-term ties that permits changes in lifestyle, role and residence; (4) a time of adaptive self-focus as young people attempt to invest in their own future; and (5) a time of future-focus and optimism (Munsey, 2006; Arnett, 2007; Arnett and Mitra, 2018).

Emerging adulthood as a purported life stage can be defined demographically too. Epidemiological data shows that most young adults in Western countries now choose to wait for a decade or more after turning 18 before having children, or before starting a marriage or civil partnership (Perelli-Harris and Lyons-Amos, 2015). This demographic fact of postponing parenthood and marriage is integral to the theory of emerging adulthood, as it is during the years prior to settling down that young adults can continue the exploration of their identity, roles and relationships, via the accrual of new and diverse life experiences (Arnett, 2000). Questions have been raised over whether or not the theory of emerging adulthood applies to individuals or subcultures who still settled down at the age of 18 or soon after (Nelson and Barry, 2005). Yet the theory pertains to what is normative rather than universal, hence accepts that there will be minorities that do not fit the model. There is now a substantial body of research attests to its wide generalizability across socio-economic groups and cultures at a normative level (Arnett, 2016; Robinson, 2016). However, the theory is also clear that economic and cultural contexts frame the experience of emerging adulthood, and hence research on the topic should be explicit in its acknowledgment of cultural context (Konstam, 2007). The current study focuses on the United Kingdom and the United States, two countries in which rates of tertiary education are relatively high and young adults must pay for such education, typically leading to large debts. There is evidence to suggest that levels of stress are high in emerging adults in the United Kingdom and the United States compared with other age groups within each country (Stone et al., 2010; Forth, 2018).

Early adult crisis episodes typically occur toward the latter end of the life stage of emerging adulthood, and last approximately a year (Robinson, 2016). They are periods of instability, transition and heightened emotion, and are typically triggered when a person makes substantive and active efforts to settle down into a steady set of life roles but then experiences difficulties that lead to feeling overwhelmed and unable to cope (Robinson, 2016). As such, early adult crisis typically revolves around a struggle with either feeling ***locked out*** of adult commitments (being unable to find work or love), or the feeling of being ***locked in*** to life roles that are then experienced as a poor fit for one’s identity, or as generally stultifying (Robinson, 2016, 2019). During an early adult crisis, there are reports of strong negative emotions but also of enhanced curiosity and opportunities for growth and change (Robinson et al., 2017). There is also an intensive focus on the question of personal and social identity, with people who are in crisis during this age group engaging in a process of questioning ‘who I am’ in the context of the roles and relationships, and making active attempts to answer such questions through exploration and trial-and-error (Robinson et al., 2013). Following an early adult crisis, there may be post-crisis growth if substantive lifestyle changes are made, or feelings of depression and lowered self-esteem if attempts to cope fall short (Robinson and Wright, 2013). Early adult crises are widespread; in a United Kingdom sample, 70% of people in their 30s report that they had one in their 20s (Robinson and Wright, 2013).

Based on a review of popular definitions of QLC and the model of early adult crisis, Robinson (2016) concluded that they can be considered essentially synonymous. Counselors and coaches frequently use the term QLC to frame transitional difficulties and concomitant emotional challenges that young adults experience (Stapleton, 2012; Hapke, 2017). It has also become a topic of discussion in popular self-help literature and the media (Jay, 2012; Rosen, 2019). Given this widespread general usage, it is unsurprising that the phrase has also become part of the vernacular of many young adults who attempt to make sense of their personal challenges through the lens of QLC. We assume that given the relatively loose meanings surrounding the construct in the popular domain, references to QLC in social media are likely to be shorthand for many issues that pertain to being a young adult more generally, and so will not only link to early adult crisis, but also to many of the issues that pertain to emerging adulthood, such as uncertainty, stress, self-focus, and feeling caught in-between (Black, 2010).

The portrayal of autobiographical events and experiences on social media is an important frontier for psychological research, showing promise as a tool for studying lifespan development and mental health (Toseeb and Inkster, 2015). Facebook postings, tweets, emails and text messages that contain information about ongoing personal experiences and life events can collectively be referred to as micro-narratives (Giles, 2017), or small stories (Georgakopoulou, 2017). Each of these brief texts tells a story to an intended audience, while drawing on generic constructs that link each posting to broader cultural concerns or popular academic theories (van Dijck, 2007). Social media postings that relate to actual life events and experiences can be argued to serve a developmental function, which is to represent and reify the passing of time into a simplified and publicly documented life story that can help the individual create a meaningful ongoing narrative of how their life is changing (Rettberg, 2009).

In terms of analyzing social media data, developing vectoral representations for words (i.e., word clouds) using AI machine learning systems such as Word2Vec has recently been gaining popularity as a way of representing language usage (Mikolov et al., 2013b). These methods permit the capture of local context order rather than just “bag-of-words” relatedness, which in turn leads to the capture of syntactic information.

The aim of this study was to explore if QLC is represented in social media using linguistic features that can provide empirical illuminations about emerging adulthood and early adult crisis within the context of the United States and United Kingdom. This study is, to the best of our knowledge, the first attempt to study the language of QLC through the application of natural language processing on social media data. Twitter was selected as the social media platform on which to investigate this phenomenon for two reasons. Firstly, tweets are public and searchable, unless the user opts out by making them private. In contrast, posts on other social media platforms are mainly restricted to a defined audience and not publicly searchable. Secondly, it has been shown to be a very conducive platform for self-disclosure related to a wide variety of phenomena such as personality, stress, and other mental health categories (Coppersmith et al., 2014; Guntuku et al., 2019a, b).

A skip-gram AI model with negative sampling (Mikolov et al., 2013a) was originally used to learn word embeddings from a corpus of 400 million tweets (Lampos et al., 2014). This same method has been shown to successfully predict the income (Lampos et al., 2014) and personality of Twitter users (Guntuku et al., 2017a).

We hypothesized that the topics discussed by users who expressed having a QLC would significantly differ from a matched control group who have not used this term, in ways that support existing theory on emerging adulthood and early adult crisis. An open vocabulary analysis approach was conducted in which key QLC topics were ascertained by clustering co-occurring tweets. Given its open and exploratory focus, no word-specific or term-specific predictions were made for this analysis.

For the second analysis, run via the Linguistic Inquiry Word Count (LIWC) system, we predicted, based on emerging adulthood theory, that movement-based words (reflecting exploration), negative emotion words (reflecting instability) and personal pronouns (reflecting self focus) would be more prevalent in the QLC group compared to the control group.

A third analysis was conducted on twenty terms selected to represent early adult crisis theory, based on a conceptual and thematic review of qualitative studies on early adult crisis (*Stuck; Trying; Leave; Change; Unemployed; Lonely; Hopeless; Overwhelmed; Unfair; Fail; Coping; Failing; Debt; Meaning; Trapped; Try; New; Identity; Sacked; Money).* We predicted that these would all be more prevalent in the QLC group than the control group.

## Materials and Methods

### Participants

Data used for this study were derived from public messages posted on Twitter from 2011 to 2015. Using Twitter Search API^1^, we obtained a set of 3,200 unique users aged 18–30 from the United Kingdom and the United States who mentioned having a QLC. Tweets were filtered to deselect any retweets, URLs, advertisements and spam. Tweets with reference to ‘Happy Birthday’ were also removed to help avoid ironic mentions of QLC. Users were also filtered out if they did not have over 40 messages to ensure there would be enough history and activity to analyze. After further validation of the number of posts to obtain reliable language-based estimates, there were 1,390 users. For each user, we obtained their entire timeline of Tweets (maximum of 3,200) from Twitter API resulting in over 1.5 million messages across all users. These users (the QLC group) were matched with a control sample of Twitter users, who never mentioned having a QLC, consisting of the same age and gender distribution and who had posts around the same time period as the QLC group. The mean age of the QLC and control groups was 23.95, and the standard deviation was 2.74. Each groups contained 1,195 females and 195 males. The high percentage of females in the groups is congruent with previous findings that (a) more females than males self-report early adult crises (Robinson and Wright, 2013), and (b) women discuss emotional matters on Twitter more than men do (Kivran-Swaine et al., 2012).

Table 1 highlights the process of data collection (Table 1A) and the composition of the data (Table 1B).

**TABLE 1A:** Process on participant recruitment and selection.

Select public Twitter messages for users that used quarterlife crisis in their messages (messages ∼ 130,000)
—
↓
Manually verified the authenticity of the tweets and filtering out any non-English retweets, urls, or birthday references. (n = 3200)
—
↓
Filter users who had minimum amount of messages (>40) and maximum age of 30 to support an appropriate analysis(n = 1390)
—
↓
Matched with control group based on age, gender, and timeline. This provided over 1 million messages to be input for the language and user trait analysis.

**TABLE 1B:** Data set of control and QLC groups – gender and mean age.

Group	Count	Male	Female	Mean age	Standard deviation
Control	1390	195	1195	23.95	2.74
QLC Group	1390	195	1195	23.95	2.74
Total	2780	390 (14%)	2390 (86%)	23.95	2.74

### Linguistic Analysis

We used three sets of language analysis: (a) Open-vocabulary clustering (b) Linguistic Inquiry Word Count (LIWC) analysis (c) Theory-based analysis. These language features have been shown to be predictive of several health outcomes, such as depression, schizophrenia, attention deficit hyperactivity disorder (ADHD), personality, and general well-being (Schwartz et al., 2013, 2016; Guntuku et al., 2017b, c).

#### Open-Vocabulary Approach

An open-vocabulary statistical learning and modeling approach was used to find topics that the QLC group talk about more than the control group. This was conducted using an open source language analysis toolkit (DLATK) (Schwartz et al., 2017). From each post, words were identified (using an emoticon-aware tokenizer which also looked for tokens such as ‘:)’, ‘:-D’ etc.) and multi-word expressions were selected, keeping 2- and 3-grams (two or three consecutive words) with the highest pointwise mutual information (PMI) or association between their words. PMI is the ratio of the joint-probability to the independent probability of observing the phrase:

$$p\,m\,i\,\left(p\,h\,r\,a\,s\,e\right)=\log\frac{p\,\left(p\,h\,r\,a\,s\,e\right)}{\prod_{w\,\varepsilon \,p\,h\,r\,a\,s\,e}p\,\left(w\right)}$$

In practice, we kept phrases with PMI values greater than 2 ^∗^ length, where length is the number of words contained in the phrase, to ensure retained phrases were informative parts of speech and not just accidental juxtapositions. All word and phrase counts were normalized by each subject’s total word use [p(word j subject)], and we applied the Anscombe transformation, where vocab(subject) returns a list of all words and phrases used by that subject. These Anscombe transformed “relative frequencies” of words or phrases (p_a__ns_) were then used as the independent variables in all our analyses.

$$p\,\left(p\,h\,r\,a\,s\,e|s\,u\,b\,j\,e\,c\,t\right)=\frac{f\,r\,e\,q\,\left(p\,h\,r\,a\,s\,e,s\,u\,b\,j\,e\,c\,t\right)}{\sum_{p\,h\,r\,a\,s\,e^{\prime }\,\varepsilon \,v\,o\,c\,a\,b\,\left(s\,u\,b\,j\,e\,c\,t\right)}f\,r\,e\,q\,\left(p\,h\,r\,a\,s\,e^{\prime },s\,u\,b\,j\,e\,c\,t\right)}$$

$$p_{a\,n\,s}\,\left(p\,h\,r\,a\,s\,e|s\,u\,b\,j\,e\,c\,t\right)=2\,\sqrt{p\,\left(p\,h\,r\,a\,s\,e|s\,u\,b\,j\,e\,c\,t\right)+3\,/\,8}$$

Artificial neural networks have recently been gaining popularity because they result in low-ranking word embeddings leading to state-of-the-art results for a number of semantic tasks (Mikolov et al., 2013b). This study used a hidden layer size of 50 with the Gensim implementation.^2^ Then a spectral clustering on these embeddings was applied to obtain hard clusters of words. This resulted in 200 hard clusters, i.e., one word belongs to only one topic. The importance score associated with every word represents how central the word is in its cluster. Clusters are computed using spectral clustering over a word-word similarity matrix generated by Word2Vec. These clusters, termed as Topics in subsequent analysis, are available online.^3^

#### Linguistic Inquiry Word Count (LIWC) Analysis

This analysis consists of words grouped into 73 categories (such as Functional words, Money, Family etc.) and shown to previously predict multiple user traits such as stress, health, personality, etc. (Pennebaker et al., 2015). From each post on Twitter, we extracted the relative frequency of single words and phrases (consisting of two or three consecutive words). Then, all words used by less than 1% of users were removed from analysis so as to remove uncommonly used words (outliers). All messages used to identify the study group (i.e., tweets containing #quarterlifecrisis) were removed so that the logistic regression model captures other linguistic attributes associated with the study group above and beyond this selection criteria. The distribution of LIWC dictionary features were also extracted for each post. For each user, we measured the proportion of word tokens that fall into a given LIWC category. Then, we compared it against the word tokens from the control data using an empirical distribution of the proportion of language attributable to each LIWC category. This approach can be written out in the following way:

$$p\,\left(c\,a\,t\,e\,g\,o\,r\,y|s\,u\,b\,j\,e\,c\,t\right)=\frac{\sum_{w\,o\,r\,d\,\varepsilon \,c\,a\,t\,e\,g\,o\,r\,y}f\,r\,e\,q\,\left(w\,o\,r\,d,s\,u\,b\,j\,e\,c\,t\right)}{\sum_{w\,o\,r\,d\,\varepsilon \,v\,o\,c\,a\,b\,\left(s\,u\,b\,j\,e\,c\,t\right)}f\,r\,e\,q\,\left(w\,o\,r\,d,s\,u\,b\,j\,e\,c\,t\right)}$$

where *freq(word,subject)* is the count where the message contains the *word* and the *vocab(subject)* is the entire list of words mentioned by the subject, i.e., Twitter user.

#### Theory-Based Analysis

Based on a conceptual and thematic review of qualitative studies on early adult crisis, 20 central concepts were identified as linguistic features expected to be mentioned in social media reference to QLC (Robinson et al., 2013; Robinson, 2019). The terms associated with the QLC based on the holistic model of early adult crisis are: *Stuck; Trying; Leave; Change; Unemployed; Lonely; Hopeless; Overwhelmed; Unfair; Fail; Coping; Failing; Debt; Meaning; Trapped; Try; New; Identity; Sacked; Money.* These terms were analyzed against the data in a similar manner as LIWC (LA-b).

### Identifying Differentially Expressed Language Features During QLC

To determine if linguistic attributes (dictionary-based and open-vocabulary) and theory-based words were associated with QLC group, we individually tested them as a predictor in an in-sample logistic regression model, and report its standardized regression coefficient (β) with the associated significance. We used Bonferroni *p*-correction for multiple comparisons and use *p* < 0.05 as a heuristic for identifying potentially meaningful correlations; the effect size was measured using Cohen’s *D*. Demographic variables such as age and gender are included as covariates to obtain a unique effect of the language variables. Since we explored several features simultaneously, we consider coefficients significant if they are less than a Bonferroni-corrected two-tailed *p*-value of 0.05. This sets an extremely stringent level for significance. So for example, when examining 20,000 features, in the case of words and phrases, a required *p*-value is less than 0.05 divided by 20,000 which is 2.5 × 10^–6,^ or when examining 200 topics the required *p*-value is less than 2.5 × 10^–4^, and when examining 73 LIWC categories *p*-value is less than 6.8 × 10^–4^.

## Results

### Open-Vocabulary *Clustering* Approach

**FIGURE 1:**
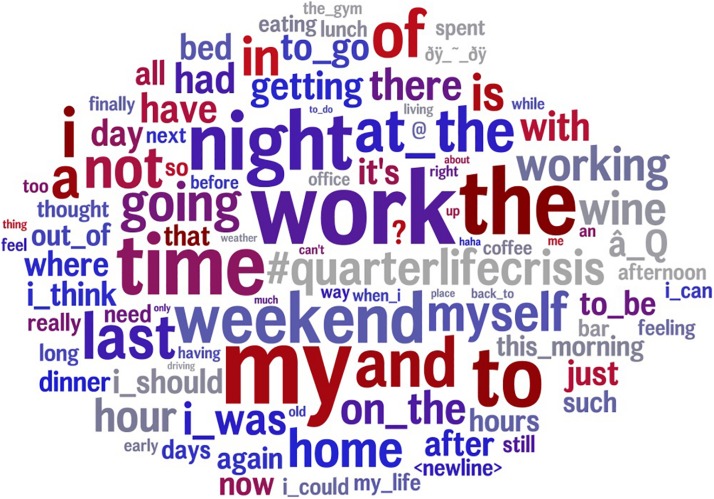
The most frequently used words for those who describe themselves as going through a quarter-life crisis. Word size indicates the strength of the correlation to QLC and word color indicates relative word frequency – red is more frequent, and gray is less frequent (*p* < 0.05, Bonferroni *p*-corrected).

Based on the open-vocabulary analysis using vector-based graphic representations of term clusters that correlated with QLC, Figure 1 shows the most prominent words and phrases in the Twitter messages posted by the QLC group compared with the control group. In the figure, word size represents the strength of the correlation to QLC and word color indicates relative word frequency.

Words relating to time (“night”; “weekend”; “morning”; “early”; “day”) and work (“work”; “working”) had the highest frequency and correlation strengths. Also, a pronounced use of first person pronouns (“I”; “my”; “myself”) was observed in users going through QLC. References focused on reflection and a willingness to conduct activities (“i_should”, “i_could”, “i_can”) have been identified.

**FIGURE 2:**
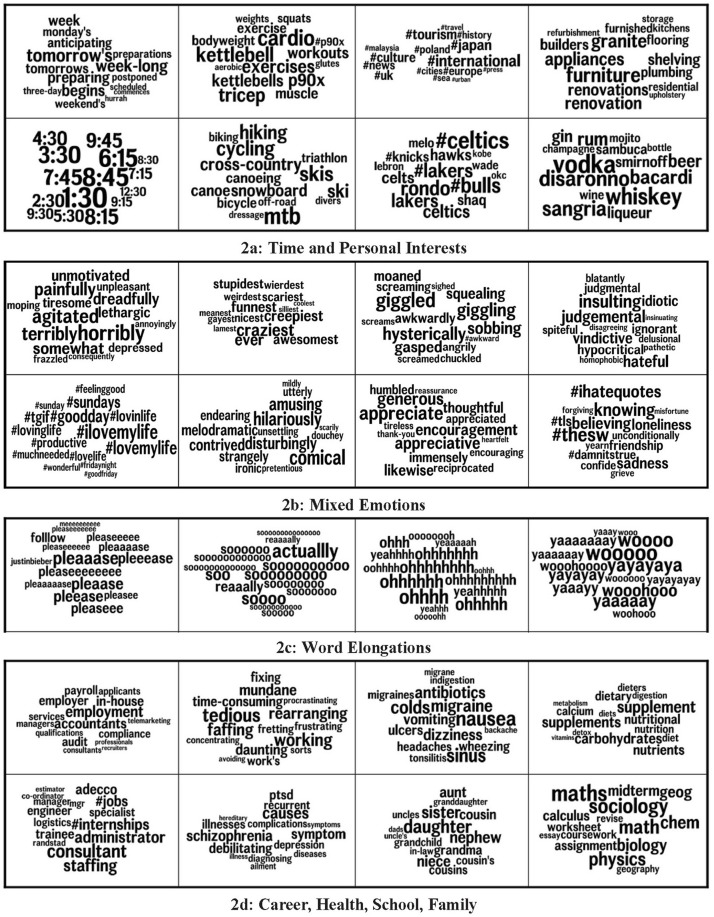
Highly correlated topics (grouped into categories) significantly associated with quarter-life crisis at *p* < 0.05 after Bonferroni *p*-correction. **(a)** General Topics (Time, Exercise, Travel, Entertainment). **(b)** Mixed Emotions. **(c)** Elongation. **(d)** Career, Health, School, Family.

Figure 2 shows topics that are associated with QLC. Each individual box represents an output from the Word2Vec vector analysis. These were grouped into four thematic categories by the authors for the purposes of parsimonious presentation. Every topic in each category was significantly associated with QLC at *p* < 0.05 after Bonferroni *p*-correction. Figure 2a highlights topics of everyday life including issues of timing, exercise, fitness, traveling, sports, domestic settling down, and alcohol. Figure 2b indicates the emotional dysfunction that accompanies the strife and confusion of QLC. The range of emotions extends from positive (“awesomest”, “\#ilovemylife”) to negative (“sadness”, “agitated”) sentiments. In addition to expressing themselves through emotional words, the QLC group tends to emote through elongated words (Figure 2c). Elongation is common in social media and provides nuance to digital communications by mimicking intonation found in vocal exchanges (Doll, 2013). A final cluster of topics (Figure 2d) includes lifestyle and health issues, including employment, mental health problems, physical illness, family, food and studying.

### Linguistic Inquiry Word Count (LIWC) Analysis

LIWC conducts all inferential analysis using Cohen’s d. Effect size between QLC and LIWC linguistic categories are shown in Table 2, grouped into categories for readability. Categories that were significant at *p* < 0.05 after Bonferroni correction are shown in the table. Positive effect size represents an association between the category and the presence of QLC.

**TABLE 2:** Results from LIWC Categories significant after Bonferroni *p*-correction.

LIWC category name	Sample words	Cohen *d*
**Relativity**		
Time	When, Now, New	1.317
Space	In, On, Out	1.080
Motion	Go, Going, Come	0.718
**Time Orientations**		
Present Focus	Is, Have, Are	0.928
Future Focus	Will, Then, Gonna	0.789
Past Focus	Was, Got, Been	0.779
**Biological Process**		
Ingestion	Eat, Sweet, Drunk	0.958
Health	Sick, Tired, Living	0.713
**Cognitive Processes**		
Insight	Know, Think, Find	0.842
Differentiation	If, Or, Can’t	0.671
Certainty	All, Never, Ever	0.592
Discrepancy	Want, Need, Would	0.568
Tentative	Same, Hope, Any	0.501
Causation	How, Why, Make	0.473
**Other Grammar**		
Comparisons	Like, As, Than	0.747
Quantifiers	More, Some, Much	0.794
Common Adjectives	As, More, New	0.792
Interrogatives	What, How, Who	0.583
**Perceptual Processes**		
Feel	Feel, Hard, Feeling	0.67
Personal Concerns		
Home	Home, Bed, House	0.850
Leisure	Fun, Twitter, Play	0.475
Work	Work, School, Class	0.432

**Psychological Processes**		
***Negative Emotion***		
Anxiety	Awkward, Worry, Scared	0.631
Sadness	Miss, Sorry, Sad	0.501
Risk	Bad, Stop, Wrong	0.453
**Informal Language**		
Non-fluencies	Oh, Well, Ugh	0.534

**Linguistic Dimensions**		
***Function Words***		
Common Adverbs	So, Just, When	1.164
Prepositions	To, Of, In	1.145
Articles	The, A, An	1.005
Auxiliary Verbs	Is, Be, I’m	0.974
Conjunctions	And, So, But	0.827
Impersonal Pronouns	It, This, That	0.792
1st Pers Singular	I, My, Me	0.774
Negations	Not, Don’t, No	0.559

For QLC individuals, Time (*d* = 1.317) had the largest effect size LIWC category followed by Space (*d* = 1.080) and Motion (*d* = 0.718) under Relativity, potentially indicating the phases of transition with school, career, and locations in emerging adults. Replicating previous findings (Jay, 2012), we found a high effect size with Present Focus (*d* = 0.928) relating to the present bias of QLC individuals. The large effect sizes associated with Biological Processes, specifically Ingestion (*d* = 0.958) and Health (*d* = 0.713) aligns with some of the previously identified common pressure pain points associated with QLC (Panchal and Jackson, 2007). QLC individuals also tend to use language associated with Cognitive Processes, including Certainty (*d* = 0.592), Discrepancy (*d* = 0.568), Tentativeness (*d* = 0.501), indicative of mixed reactions and emotions. Similar observations can be made about QLC’s association with grammatical categories such as Comparisons (*d* = 0.747) and Interrogatives (*d* = 0.583). As a part of emerging adulthood, individuals consider where to live, how to live, and what to do, consequently producing language associated with Personal Concerns, specifically Home (*d* = 0.850), Leisure (*d* = 0.475), Work (*d* = 0.432), and Feelings (*d* = 0.676). Language of QLC individuals has a high association with Anxiety (*d* = 0.631) and Sadness (*d* = 0.501), and Risk (*d* = 0.453). Other effect size with Non-fluencies (*d* = 0.534) and Function words (e.g., 1st person pronouns, *d* = 0.774) are in line with the results from the open vocabulary approach.

### Theory-Based Analysis

In order to test the hypothesis that the 20 selected theoretical terms would be more prevalent in the QLC group than the control group, a language analysis was conducted using the same method as LIWC. The results in Table 3 show which theoretically derived terms were significantly higher for the QLC group than the control group. Sixteen of the twenty terms were significantly different across QLC and control group, providing robust evidence that discussions of crisis on Twitter show a linguistic fit with what is known about crisis in young adults from a theoretical and empirical standpoint.

**TABLE 3:** Common Words associated with quarter-life crisis in the Twitter dataset.

					Confidence interval		
Rank	Label	*d*	*p*	*	Lower	Upper	Frequency
1	Stuck	0.376	<0.001	*	0.149	0.22	5220
2	Trying	0.339	<0.001	*	0.131	0.203	22990
3	Leave	0.330	<0.001	*	0.127	0.198	13316
4	Change	0.173	<0.001	*	0.049	0.122	12531
5	Unemployed	0.158	<0.001	*	0.043	0.116	256
6	Lonely	0.158	<0.001	*	0.042	0.115	1678
7	Hopeless	0.144	<0.001	*	0.036	0.109	317
8	Overwhelmed	0.126	<0.001	*	0.026	0.1	530
9	Unfair	0.106	0.01	*	0.021	0.094	415
10	Fail	0.112	0.01	*	0.02	0.093	2642
11	Coping	0.102	0.01	*	0.015	0.088	105
12	Failing	0.100	0.01	*	0.013	0.087	635
13	Debt	0.096	0.01	*	0.012	0.085	552
14	Meaning	0.096	0.01	*	0.011	0.085	1399
15	Trapped	0.086	0.03	*	0.006	0.08	584
16	Try	–0.104	0.01	*	–0.088	–0.015	18692
17	New	–0.070	0.08		–0.072	0.001	75993
18	Identify	0.068	0.09		–0.003	0.07	564
19	Sacked	0.064	0.1		–0.005	0.069	84
20	Money	0.772	0.36		–0.055	0.018	13649

* Significance *p* < 0.05.

## Discussion

This study leveraged social media data to uncover the online word usage of Twitter users from the United Kingdom and the United States who report experiencing a QLC. Using an open vocabulary AI-based clustering method, we hypothesized that we would find differences between those reporting QLC and matched non-QLC control group. This was supported, and the results shown in Figures 1, 2 show clear links with the theory of emerging adulthood and the holistic model of early adult crisis. The most common words in Figure 1 were *work, time, night, weekend* and *my*. *Work* was the word most strongly associated with QLC. This fits with the fact that accounts of QLC mainly revolve around problems with finding, or adapting to, work. In a large quantitative survey of crisis features, the most prevalent features in early adult crisis amongst men were “Feeling trapped in a job you didn’t want to be in any more,” and “Experiencing a high level of stress and pressure in your job” (Robinson and Wright, 2013). These features were also very prevalent amongst women. The association of the word *time* with QLC is illuminated by the top right cluster in Figure 2, which shows a future focus, with words like *tomorrow, preparing* and *anticipating*. This fits with one of the five core features of emerging adulthood, which is an optimistic preoccupation with the future. With regards to the use of first person pronouns in QLC tweets relative to the control (*my, myself, I*) as shown in Figure 1, this fits with previous findings showing a heightened use of personal pronouns on social media by users with mental health issues relative to a control (De Choudhury et al., 2013). It also fits with the theoretical postulates of both emerging adulthood and the model of early adult crisis. The former suggests that young people who are passing through emerging adulthood have a relatively strong self-focus compared with adults of other age groups (Arnett, 2000). If QLC is partly used in social media as a proxy for the challenges of emerging adulthood, as we have hypothesized, one would expect a high level of usage of personal pronouns in the social media language of QLC. The use of personal pronouns fits with early adult crisis theory insofar as the latter purports that crises involve an extended questioning of identity, in terms of ‘who *I* am’ in the context of society, roles and relationships (Robinson et al., 2013).

The topics represented in Figure 2a – exercise, travel, alcohol, sport, time - reflect many of the topics that emerging adults engage in to both cope with stress and find meaning (Arnett, 2014). The time-related words in the top right box of Figure 2a include mainly future-focused terms, such as tomorrow, preparations and anticipating, as previously discussed. Exercise and fitness are effective ways of managing stress, so may be linked to QLC as coping strategies (Cairney et al., 2014). On the flipside, alcohol usage is also linked to QLC. Epidemiological research shows that alcohol consumption peaks in emerging adulthood (McManus et al., 2016), and it has been theorized that this may be a form of self-medication for stress and anxiety (Cooper et al., 1992). The cluster of terms on tourism and traveling relates to the phase of exploration in the holistic model of QLC, which often involves taking time-out from long-term commitments to go abroad, with the aim of getting perspective on one’s current life circumstances and priorities (Robinson et al., 2013). The cluster of terms on domestic furnishing fits with the fact that early adult crisis tends to occur just as a young adult is making active attempts to settle down and develop a stable lifestyle (Robinson, 2016). Many of these topics that associate with QLC are likely to be framed by cultural factors of the United Kingdom and the United States, for example the relative affluence and high employment rates of these countries, the relatively high tertiary education participation, the high stress levels in young adults (Stone et al., 2010; Forth, 2018). The potential generalization of the current study that we tentatively claim is to young adults within these two countries. While QLC is a phenomenon that is discussed in other cultures such as India and China (e.g., Mehta, 2008; Mei, 2017), the linguistic associations of the phenomenon in these other countries may differ substantively.

Social media captures users’ emotions in an ecological and relatively immediate setting (Suler, 2004). Our finding that expressing *mixed* emotions (Figure 2b) associates with QLC supports the affective strand of the holistic theory of early adult crisis, which represents emotions as during a crisis episode as a combination of emotional conflict and negativity but also times of excitement, hope, and fun (Robinson et al., 2013). The same mix of positivity and negativity has been found in other previous studies of QLC (Panchal and Jackson, 2007; Black, 2010; Robinson, 2019). Furthermore, employment and family (Figure 2d) have been identified as pressure points during transitions in emerging adulthood (Panchal and Jackson, 2007). The cluster of mental health terms in Figure 2d fits with the acknowledged overlap between QLC and mental illness, such that periods of crisis are times of heightened vulnerability for mental illness, particularly if the individual does not enact changes that permit resolution of the crisis, so the difficulties and instabilities associated with the crises become chronic (Robinson, 2016).

QLC episodes are periods of high intensity experience, in terms of major decisions to take, challenging problems to surmount, and strong emotions to manage. A novel finding from the study is the association between QLC and word elongations such as *meeeee, pleaseeeee, yeaahhhhh, reeaallly, soooo*, and *yaaaaaaay*. We propose that this form of spelling idiosyncrasy may be used to convey intensity of experience – by expanding the word in size, the strength of its meaning is enhanced to the reader. They could even be considered a marker for possible QLC – an apparently mundane linguistic quirk that may have developmental meaning. Further research could explore this in relation to other high intensity life transitions.

With regards to LIWC findings, it was found that QLC is related to words referring to time, change, and movement. This reflects how QLC is often a time of transitional change and active movement. It was also found that QLC is associated with being focused on both the present moment and the future, reflecting how immediate concerns to cope with and struggle against pressing challenges draw attention to the present moment, and also to the question of where life is going. This finding reflects work by Jay (2012) on the ‘present bias’ of young adults more generally – such a bias may be amplified in times of QLC. The association with words about eating and health is likely to refer to concerns about well-being, stress and health that have been regularly found to precipitate a developmental crisis (Robinson, 2016) or create a sense of existential concern in young adults (Panchal and Jackson, 2007). The other key categories; insight, feeling, home, and anxiety also reflect various facets of what is known about QLC. Anxiety and feeling words reflect the strong affective content of crisis episodes; insight reflects the heightened curiosity and questioning that has been found to be present in crisis episodes (Robinson et al., 2017); home reflects the central issue in QLC of ‘where I fit in’ to the world and where one will end up living as an adult (Robinson, 2019).

The theory-led term analysis shown in Table 3 strongly supports the proposition that QLC links to the theoretical model of early adult crisis (Robinson, 2016). Sixteen of the twenty terms predicted to associate with QLC from this theory (*stuck, trying, leave, change, unemployed, lonely, hopeless, overwhelmed, unfair, fail, coping, failing, dept, meaning, trapped* and *try*) did indeed link to Twitter postings about it. The four words that did not were *new, identify, sacked* and *money*. Our interpretations of the absence of these four are speculative. It may be being *sacked* is more likely to be a feature of crisis in midlife or that *fired* is a more common term of use among young adults, and while debt is a clear feature of crisis, *money* was not associated perhaps because of its value neutrality. *Identify* may be too abstract a verb for Twitter postings, and *new* may be used in too wide a range of ways to make it a crisis differentiator.

Our use of data from Twitter has introduced a new lens on the analysis of the QLC and how the challenges of being a young adult are discussed on social media. There are however various limitations to this study. The individuals who post on Twitter may not represent a full range of socioeconomic status groups or may be systematically different from the general population in other unspecified ways. Hence, those who post about QLC on Twitter may not represent those who would report having QLC in the general population. However, given the naturalistic conditions of data collection and the relatively large sample of this study, it is arguably more likely to generalize than most surveys. We do not assume that QLC is a phrase used in other languages, so we do not generalize these findings to non-English-speaking countries. In addition, the study filtered for English-only tweets, irrespective of cultural origination, which could have introduced cultural confounds in language.

Finally, while we have framed our interpretation of the results through the theory of emerging adulthood and the model of early adult crisis, we do not have the scope here to systematically compare these with other potential theories as interpretative frameworks. We claim a good fit between data and theory in a host of ways, but other theories may also provide helpful abductive schemes.

Notwithstanding these limitations, this is the first academic study known to use artificial intelligence and social media to study the discourse surrounding QLC at scale. Theory on emerging adulthood and early adult crisis has not previously made use of Big Data to test theoretical postulates and explore new areas. We argue that the study provides a new empirical lens on the developmental challenges that young adults experience and the language used to frame experiences on social media. Important next steps for further research include exploring how the link between QLC and language is moderated by gender, age, geographical location and ethnicity. Another option for future studies includes using the same methodology to analyze midlife crisis and later life crisis. Finally, a longitudinal analysis would be revealing of how a mention of QLC predicts social media postings over time. It would be possible to take a group of individuals who mention QLC on Twitter for the first time and then explore the contents of their postings at several time points (e.g., 6 months later and a year later) relative to a matched control group. As well as a stimulus for more research, the study has a number of possible practical applications.

Following further studies to ensure replicability and a fine-grained understanding of gender and culture, we intend to develop a guide for clinicians, coaches and university lecturers in the United Kingdom and United States to help understand how young adults verbally discuss their personal challenges with each other in the space of social media, and what kinds of issues and words used in tweets may be indicative of a personal crisis and hence in need of targeted support and help.

## Data Availability Statement

The datasets for this manuscript are not publicly available to protect the privacy of the users. Requests to access the dataset should be directed to SG, sharathg@sas.upenn.edu.

## Ethics Statement

The studies involving human participants were reviewed and approved by University of Pennsylvania. Written informed consent for participation was not required for this study in accordance with the national legislation and the institutional requirements.

## Author Contributions

SA and SG originated the study. SA, SG, OR, and LU developed the methods, interpreted the analysis, and contributed to the writing of the manuscript. AD assisted with critical developments to the manuscript, including theoretical content.

## Conflict of Interest

The authors declare that the research was conducted in the absence of any commercial or financial relationships that could be construed as a potential conflict of interest.

## Footnotes

1 https://twitter.com/search-home

2 https://radimrehurek.com/gensim/

3 https://web.sas.upenn.edu/danielpr/resources
